# Caution

**Published:** 1889-09

**Authors:** 


CAUTION!—Dr. C. H. Worrick writes: In the SOUTHERN MEDICAL RECORD for July, page 264, Dr. J. B. Johnson gives a prescription in which twelve grs. of potassa chlorate is given to a child every half hour, with favorable results.

Now M. Brouardel cites six cases of death resulting from four grain doses of the same medicine administered as stated above. He adds: "Physicians on account of the scant information obtained as to the physiological action of chlorate potassium use the medicament with the same imprudence and indifference as they have formerly shown."

